# Intra-axial Neurenteric Cyst of Medulla: Case Report and Literature Review

**DOI:** 10.7759/cureus.15361

**Published:** 2021-05-31

**Authors:** Andrey V Gavrjushin, Danil M Chelushkin

**Affiliations:** 1 7th Neurosurgical Department, N. N. Burdenko National Medical Research Center of Neurosurgery, Moscow, RUS; 2 9th Neurosurgical Department, N. N. Burdenko National Medical Research Center of Neurosurgery, Moscow, RUS

**Keywords:** intra-axial cyst, cyst of medulla, neurenteric cyst

## Abstract

Neurenteric cysts (NCs) are rare congenital lesions that are thought to result from the persistence of the neurenteric canal connecting primitive gut and neural tube. Despite the congenital nature, NCs can be diagnosed at any age and at a similar frequency in women and men. To our knowledge, 140 intracranial NCs, confirmed by histology, including the patient presented in this review, have been reported since 1952. Parenchymal NCs are extremely rare, and there are no publications describing the intra-axial NCs of the brainstem at the moment.

A 19-year-old female presented to the clinic with the following complaints: moderate dysphagia (two to three times per day) for and liquids and solids, dysphonia, vertigo, spontaneous nystagmus, imbalance, and numbness in the left side of the body, including the face. The magnetic resonance imaging (MRI) of the brain showed a well-defined lesion centered in the medulla. The patient underwent a small right-sided keyhole retro-sigmoid craniotomy. Just under the sulcus, a cyst containing pathological amorphous gray-yellow liquid was evacuated. Accessible parts of the capsule were resected without brain injury. Residual particles of the capsule were coagulated.

Two months after the operation, the patient presented with similar symptoms. We used the previous craniotomy during the second surgery. After the evacuation of the cyst, a silicone stent was set for connecting with the cerebellopontine cistern and preventing new synechiae formation. As surely as after the first surgery, all neurological symptoms gradually regressed. In two months after surgery, deglutition and sensations recovered, and hemiparesis and imbalance decreased. Postoperative MRI examination two months after surgery showed no evidence of cyst recurrence.

Intra-axial NCs are a rare group of congenital pathological lesions with a favorable prognosis. There are no publications of brainstem NCs with intra-axial localization to date. The treatment of choice in this group of patients is complete microsurgical excision followed by long-term observation.

## Introduction

Neurenteric cyst (NC) was first described in 1928 by Kubie [1] and in 1934 by Pussep [2]. Since then, it is known as enterogenic cyst, endodermal sinus cyst, intestinal cyst, teratomatous cyst, intestinoma, and archenteric cyst.

Neurenteric cysts (NCs) are rare congenital lesions that are thought to be the result of the persistence of the neurenteric canal connecting primitive gut and neural tube. As a result of separation failure, the NC cavity canal is lined with cuboidal or columnar epithelium and contains mucin-producing cells [3-5]. Nowadays, NCs, Rathke cleft cysts, and colloid cysts belong to one general group - endodermal cysts of the central nervous system (CNS) due to their morphological similarities. This “Seessel's pouch origin” hypothesis suggesting a common origin for suprasellar NC, Rathke's cleft cysts and colloid cysts, does not seem to be perfect because it fails to explain the laterally positioned spinal neurenteric cyst (S-NC).

The other existing non-comprehensive theory includes embryonal failure of separation between the notochord and the foregut leading to the incorporation of primitive endodermal cells in the notochord. However, as the most rostral extent of the endoderm terminates at the level of the clivus, this hypothesis does not explain the occurrence of spinal NCs [6].

The most contemporary hypothesis suggests an anomalous migration of endodermal cells via the primitive neurenteric canal into the ectoderm, thus reaching far cranial and lateral locations (positions) [3-7]. According to different authors, NC comprises 0.15% to 0.35% of all intracranial lesions, 0.3% to 0.5% of spinal tumors, and approximately 16% of CNS cysts [8,9].

Despite the congenital nature, NCs can be diagnosed at any age and at a similar frequency in women and men. Compared to intraspinal NCs, which usually occur in the pediatric age groups, intracranial NCs are more common in the adult population in the third and fourth decade. The average age of patients diagnosed with intracranial NC is 34 years old [4,10,11].

Overall, 140 intracranial NCs, confirmed by histology, including the patient presented in this review, have been reported since 1952. From 1952 to 2012, a total of 139 cases of NCs were reported in the literature [8,9,12-16].

The majority of neurenteric cysts occur in the spine; they are usually ventral to the spinal cord. Intracranial neurenteric cysts are far less common. The majority of cysts are located infratentorially in the pre-pontine region, in the cerebellopontine angle, and to a lesser extent within the cisterna magna and the fourth ventricle. Parenchymal NCs are extremely rare [3,4,6,7,17], and there are no publications describing intra-axial NCs of the brainstem at the moment. For these reasons, we present our clinical case report.

## Case presentation

A 19-year-old female with complaints of hoarseness and mild dysphagia for liquids presented to the clinic; her symptoms had gradually deteriorated in the past five months. On admittance, physical examination showed moderate dysphagia (two to three times per day) for liquids and solids, dysphonia, vertigo, spontaneous nystagmus, imbalance, and numbness of the left side of the body and face.

**Figure 1:**
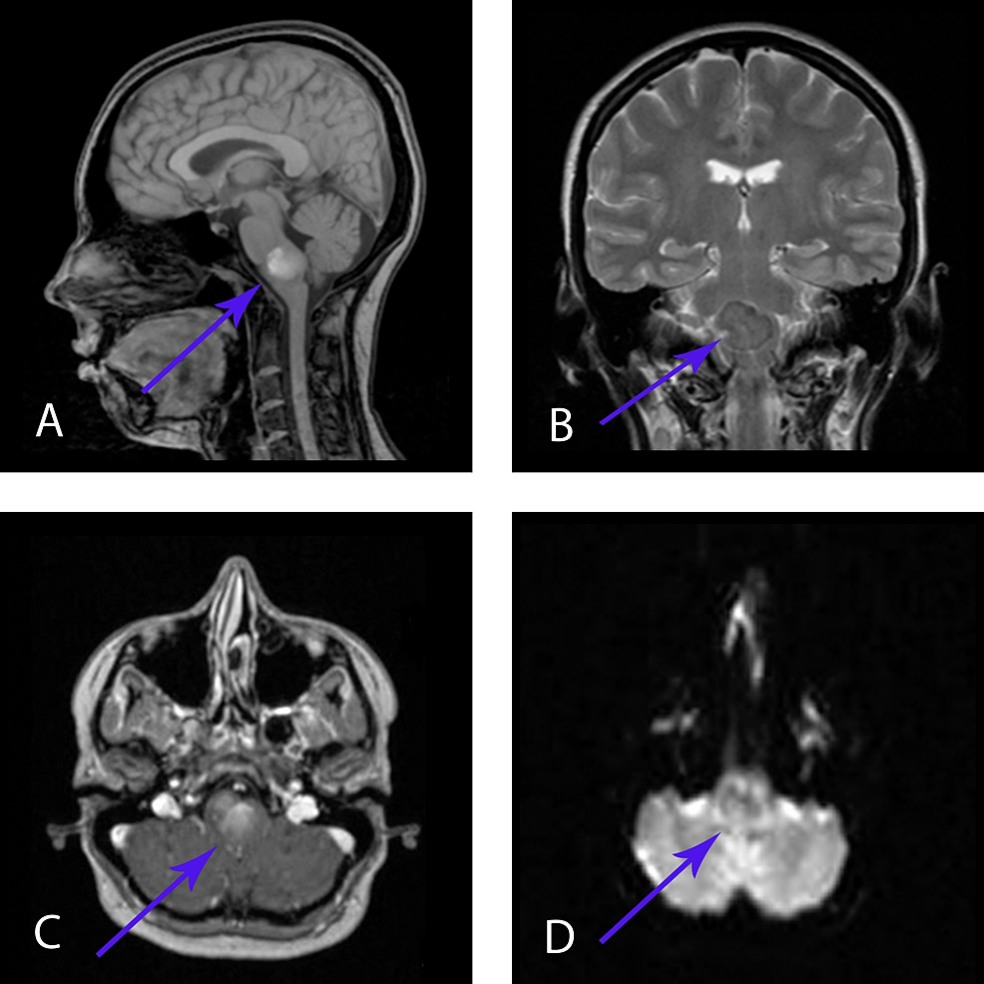
MRI, before the first surgery A – MRI Т1 sagittal view; B – MRI Т2 frontal view; C – MRI T1 + contrast, axial view; D - MRI diffusion-weighted imaging (DWI)

Magnetic resonance imaging (MRI) of the brain showed a well-defined lesion located in the medulla oblongata. This lesion was homogeneously hypointense on T2-weighted images and heterogeneously hyperintense on T1-weighted images and demonstrated no solid enhancing components. It was also heterogeneously hypointense on diffusion-weighted imaging (DWI) (Figure 1). A positron emission tomography-computed tomography (PET-CT) did not reveal any metabolic activity in this lesion. Differential diagnosis included brainstem tumor.

The patient underwent a right-sided keyhole retro-sigmoid approach in the supine position with rotation of the head to the left due to radiologic (closest medulla surface) and clinical (right-sided neurological symptoms dominance) features. Through a small craniotomy (2x1 cm) and a little C-shaped dura incision along the sigmoid sinus towards the skull base, the medulla surface was achieved. It was deformed and bulged into the сerebellopontine cistern. There were no signs of brain parenchyma destruction on the surface. So, the brainstem incision was made in a small region (almost 0.3 cm) along the retro-olivary sulcus. Just under the sulcus, the cyst containing pathological amorphous gray-yellow liquid was evacuated.

The cyst walls were presented by a thin gray capsule, tightly attached to the surrounding brain parenchyma. Accessible parts of the capsule were resected without causing any brain injury. The residual capsule fragments were coagulated. We did not perform additional endoscopic revision of the cyst cavity due to its surgical accessibility. We used transcranial motor evoked potentials and direct caudal brainstem mapping during the operation.

Numbness of the left side of the body and all clinical signs from posterior cranial fossa (PCF) increased after surgery while bulbar disturbances considerably decreased. Control computed tomography (CT) demonstrated decreased deformity of the caudal brain stem parts; no cyst remnants were evident. Almost all neurological symptoms regressed in one month after the operation, including numbness. Karnofsky Scale (KS) score was 90.

**Figure 2:**
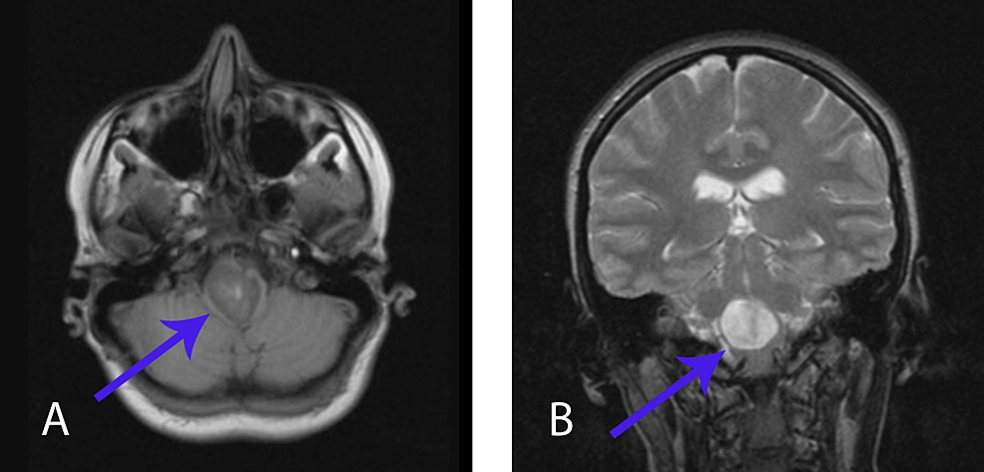
MRI, two months after the first surgery A – MRI Т1 axial view; B – MRI Т2 frontal view

In two months after surgery, the patient developed mild dysphagia and dysphonia, vertigo, spontaneous nystagmus, imbalance, and numbness of the left side of the body and the right limb, including face and mild left-sided hemiparesis before MRI was planned. MRI revealed recurrence of the cyst. As before, the lesion had well-defined, heterogeneously hyper- and isointense signal on T1-weighted images, demonstrated no solid enhancing components, but this time was homogeneously hyperintense on T2-weighted images. Sites of hyperintensity were less pronounced than on MRI before the first surgery (Figure 2).

**Figure 3:**
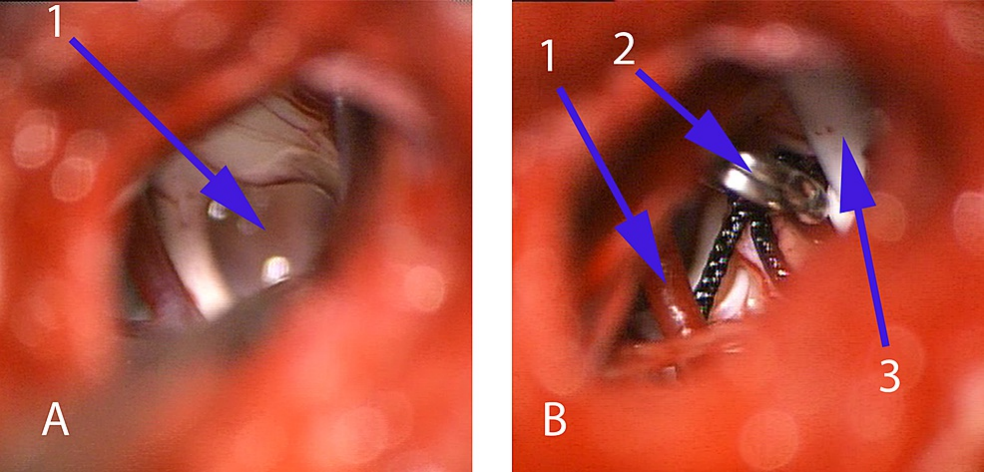
Intraoperative view during the second surgery A: view before the evacuation of the cyst on the bottom of brainstem along the retro-olivary sulcus, 1 – white viscous formless fluid in the cyst; B: view after the evacuation of the cyst, 1 – posterior inferior cerebellar artery, 2 – clip fixates stent to arachnoidea, 3 – stent in the cavity of the cyst

Repeated surgery (similar approach as in earlier surgery) showed that the site of the brainstem incision was covered with tight arachnoidal synechias. It resulted in white viscous formless fluid accumulation (Figure 3).

After the evacuation of the cyst, a silicone stent was set to connect with the cerebellopontine cistern and to prevent new synechia formation. The stent was fixated to the arachnoidea with a single clip. 

**Figure 4:**
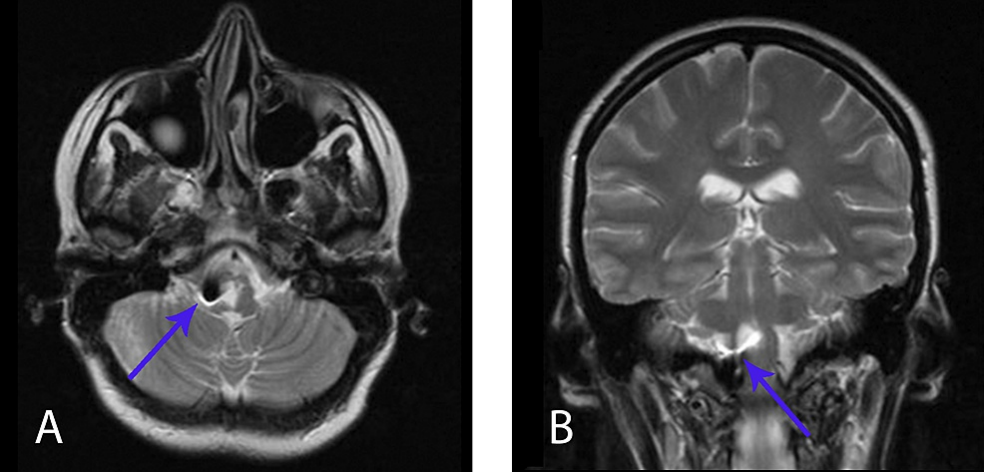
MRI, two months after the second surgery A – MRI Т2 axial view, black point – the mini-clip’s location; B – MRI Т2 frontal view, the stent in the cavity of the cyst

After the repeat surgery (as after the first surgery), all neurological symptoms gradually regressed. In two months after surgery, swallowing and sensibility improved (recovered) and hemiparesis and imbalance decreased. Postoperative MRI examination done two months after surgery showed no evidence of cyst recurrence (Figure 4).

Histological material after the first and second surgery was characterized by yellow-white gelatinous liquid with small parts of epithelial lining seen microscopically. The morphological appearance intraoperatively and histological findings matched with features of NC.

## Discussion

NC is a rare congenital abnormality of the CNS, most likely localized in the inferior cervical and superior thoracic parts of the spinal canal. Most intracranial NCs are localized in PCF in basal cisterns, craniocervical junction, or 4th ventricle. Parenchymal localization is extremely rare [3,4,6,7,17].

MRI examination is a method of choice for diagnosing this type of pathology, but its characteristics can be variable. It shows well-demarcated lesions, which are iso- or tightly hypointense on T1 and hyperintense on T2-weighted sequences and fluid attenuation inversion recovery (FLAIR) sequence [3,4,6,17]. NCs usually demonstrate no contrast enhancement, but Prasad et al. reported a mild posterior rim enhancement that, in his opinion, can be explained by chronic inflammatory changes due to repeated cyst rupture [18]. In our case, MRI characteristics were not typical. Before the first surgery, the lesion was heterogeneously hyperintense on T1-weighted images and homogeneously hypointense on T2-weighted images (Figure 1). Before the second surgery, MRI characteristics changed. It was hyperintense on T2, and sites (areas) of hyperintensity on T1 were less pronounced than before the first surgery (Figure 2), which was most likely for NCs. Such a change of the signal can be explained by a high concentration in the liquid cyst cavity. It led to heterogeneously hyperintense signal on T1-weighted images and homogeneously hypointense signal on T2-weighted images before the first operation.

NCs are slow-growing benign lesions. An NC can compress surrounding neural structures during its enlargement. It leads to these clinical signs due to anatomical localization.

The treatment of choice is complete surgical excision. Clinical signs most commonly regress after surgery. Although a radical resection prevents recurrence, considering the fact that a thin capsule may firmly adhere to the brainstem, it is not always possible to completely resect the capsule without any neurological deficit, especially in PCF [4-6,17].

Kozak et al. [4] described a case series operated in the period from 2010 to 2018. There were four cases of NCs located in the superior cervical part of the spinal cord extramedullary, three cases intracranially in the craniocervical junction, 4th ventricle, and left frontal lobe. In all spinal cases, they achieved total removal. In contrast, in two intracranial cases (craniocervical junction and 4th ventricle), they were forced to leave a capsule fragment due to its firm adhesion to vessels and pia mater.

In the case series of Wang et al. [19], seven cases with NCs were described, and six of them were localized anteriorly extra-axially to the brainstem from the medulla to the C1 segment of the spinal cord and one case in the cerebellopontine angle (CPA). Gross total resection was achieved in three cases only.

Besides problems with radical resections, many authors write about the necessity of relapse surgery in cases of early NC recurrence from capsule remnants. Nelson et al. [3] present a clinical case of huge NC of CPA, 4th ventricle, and foramen Luschka. In this case, gross total resection was not achieved due to firm adhesion of the cyst’s capsule to the brainstem and cisternal segments of IX-XI cranial nerves. Later due to recurrence of NC, clinical signs progressed, and ventriculoperitoneal (VP) shunting one month after surgery and relapse surgery with a partial cyst removal in four months was required.

Chen et al. [5] showed 10 cases with NCs in their large series; five of them were spinal extramedullary and five intracranial (temporal and frontal lobe, CPA) cysts. Gross total resection was achieved in four of 10 cases. In six cases of subtotal resection, NC recurrence was marked in four cases. Relapse surgery, including resection of the recurrent cyst, VP or cyst peritoneal shunting due to worsening clinical signs were required only in three patients.

Opposed to the above described clinical case series, the reason for recurrence in our case was a firm adhesion of the capsule to the brainstem incision area, so we had to place a silicone stent in the cyst cavity for its drainage in the lateral cistern of the brain stem.

According to a literature review, NC recurrence can occur in later periods after surgery. However, the incidence of these complications is rather difficult to determine due to the rare occurrence of NCs. Only Chavda et al. indicated that incidence of NC recurrence in their series of NC subtotal resections made up 37% within a 30-year follow-up [20]. Today, many authors agree that patients with regular MRI examinations need a longer follow-up period, and repeated surgery is required if recurrence with clinical sign worsening is revealed [4].

## Conclusions

Intra-axial NC is a rare group of congenital pathological lesions with a favorable prognosis. Today, there are no publications of brainstem NCs with an intra-axial localization. The treatment of choice in this group of patients is complete microsurgical excision followed by long-term observation.

## References

[1] Kubie LS (1928). A clinical and pathological study of two teratomatous cyst of the spinal cord, containing mucous and ciliated cells. Surg Gynecol Obstet.

[2] Puusepp M (1934). Variété rare de tératome sousdural de la region cervicale (intestinome): quadriplégie, extirpation, guérison complete. Rev Neurol (Paris).

[3] Nelson SM, Mathis DA, Hobbs JK, Timpone VM (2017). Intracranial neurenteric cyst mimicking an ependymoma: imaging features, pathologic correlation and review of literature. Clin Imaging.

[4] Kozak J, Bizik I, Surkala J, Steno J, Steno A (2019). Neurenteric cysts, incidence and surgical treatment. Bratisl Lek Listy.

[5] Chen CT, Lai HY, Jung SM, Lee CY, Wu CT, Lee ST (2016). Neurenteric cyst or neuroendodermal cyst? Immunohistochemical study and pathogenesis. World Neurosurg.

[6] Góes P, Vaz-Guimaraes F, Suriano IC, Araújo S, Zymberg ST (2018). Supratentorial neurenteric cyst: analysis of 45 cases in the literature. Interdiscip Neurosurg Adv Tech Case Manag.

[7] Agrawal M, Dharanipathy S, Nakra T (2019). Supratentorial neurenteric cyst: a rare differential for a frontal cyst. World Neurosurg.

[8] Gauden AJ, Khurana VG, Tsui AE, Kaye AH (2012). Intracranial neuroenteric cysts: a concise review including an illustrative patient. J Clin Neurosci.

[9] Breshears JD, Rutkowski MJ, McDermott MW, Cha S, Tihan T, Theodosopoulos PV (2015). Surgical management of intracranial neuroenteric cysts: the UCSF experience. J Neurol Surg B Skull Base.

[10] Gu J, Yang T, Xing X (2015). A dorsally located giant posterior fossa neurenteric cyst in a Chinese woman. J Clin Neurosci.

[11] Zalatnai A (1987). Neurenteric cyst of medulla oblongata--a curiosity. Neuropediatrics.

[12] Shimizu Y, Fujita N, Akiyama O, Suzuki M, Kondo A (2019). A rare presentation of a pediatric neurenteric cyst as an intra-axial pontine lesion: a case report with a 5-year follow-up. Surg Neurol Int.

[13] Lach B, Russell N, Atack D, Benoit B (1989). Intraparenchymal epithelial (enterogenous) cyst of the medulla oblongata. Can J Neurol Sci.

[14] Agresta G, Sokol D, Kaliaperumal C, Kandasamy J, Gallo P (2020). A novel management proposal for intrinsic brainstem neurenteric cysts: case report. J Neurosurg Pediatr.

[15] Cho JM, Ahn JY, Kim SH, Lee KS, Chang JH (2010). An endodermal cyst mimicking an intra-axial tumor in the medulla oblongata. Childs Nerv Syst.

[16] Li T, Wu X, Zhang Y (2017). A rare presentation of an enterogenous cyst as an intra-axial pontine lesion. World Neurosurg.

[17] Shimanskiy VN, Shevchenko KV, Poshataev VK, Odamanov DA, Karnaukhov VV, Shishkina LV, Konovalov AN (2017). Intracranial neurenteric cysts: experience of the Burdenko Neurosurgical Institute in the XXIth century. (Article in Russian). Zh Vopr Neirokhir Im N N Burdenko.

[18] Prasad GL, Sharma BS, Mahapatra AK (2016). Ventral foramen magnum neurenteric cysts: a case series and review of literature. Neurosurg Rev.

[19] Wang L, Zhang J, Wu Z, Jia G, Zhang L, Hao S, Geng S (2011). Diagnosis and management of adult intracranial neurenteric cysts. Neurosurgery.

[20] Chavda SV, Davies AM, Cassar-Pullicino VN (1985). Enterogenous cysts of the central nervous system: a report of eight cases. Clin Radiol.

